# Psychiatric symptoms and syndromes in sarcoidosis: a systematic review and meta-analysis

**DOI:** 10.3389/fmed.2025.1634175

**Published:** 2025-09-29

**Authors:** Andreas Frans, Griet Van Hoye, Xavier Van Meerbeeck, Manuel Morrens, Filip Van Den Eede

**Affiliations:** ^1^Department of Medical Psychology and Psychiatry, Antwerp University Hospital, Antwerp, Belgium; ^2^Collaborative Antwerp Psychiatric Research Institute, University of Antwerp, Antwerp, Belgium; ^3^Department of Geriatric, Antwerp University Hospital, Antwerp, Belgium; ^4^Psychiatric Hospital Sint-Hiëronymus, Sint-Niklaas, Belgium; ^5^Department of Pneumology, Antwerp University Hospital, Antwerp, Belgium; ^6^Scientific Initiative for Neuropsychiatric and Psychopharmacological Studies, University Psychiatric Hospital Duffel, Duffel, Belgium

**Keywords:** sarcoidosis, neurosarcoidosis, depression, anxiety, fatigue, psychosis, neuroinflammatory diseases

## Abstract

**Introduction:**

Sarcoidosis is often associated with psychiatric symptoms and syndromes (PSS). This systematic review and meta-analysis aimed to summarize available literature on the prevalence of PSS in sarcoidosis, as well as their potential associations.

**Methods:**

A systematic search was conducted across PubMed, including case reports and studies that investigated PSS in sarcoidosis. A meta-analysis was performed on studies that assessed the association between sarcoidosis and PSS, using odds ratios (OR).

**Results:**

We included 43 studies and 53 cases on PSS in patients with sarcoidosis. The weighted average prevalence was 24.9% for depression, 28.7% for anxiety, 29.2% for neurocognitive symptoms, 54.4% for fatigue, 50.5% for excessive daytime sleepiness and 26.9% for sleep disturbances and insomnia, with the best available evidence for depression, anxiety and fatigue. The meta-analysis (*n* = 962) confirms that patients with sarcoidosis have a significant increased risk of developing PSS when compared with healthy controls (OR = 5.498, CI = 0.430–70.238, *p* < 0.001). Depressive symptoms and fatigue were most reported on and demonstrated the strongest associations as well (resp. OR = 4.855, z = 2.401, *p*=0.016 and OR = 20.231, z = 2.868, *p* = 0.004). Significant associations with anxiety and neurocognitive symptoms were also observed, although with less available evidence. Case reports reveal a diagnostic diversity not reflected in study populations, including psychosis and catatonia.

**Conclusion:**

Sarcoidosis is associated with a higher prevalence of PSS. Nonetheless research in this area remains limited. Systematic use of standardized psychiatric assessment tools is recommended.

## 1 Introduction

Sarcoidosis is a granulomatous multisystem disease of unknown origin with predominant lung and lymph node involvement, a heterogeneous clinical presentation and a variable clinical course (1). It is a global disease, with a prevalence of about 4.7–64 in 100,000, and an incidence of 1.0–35.5 in 100,000 per year (1). The diagnosis of sarcoidosis is based on three major criteria: a compatible clinical presentation, the finding of nonnecrotizing granulomatous inflammation in one or more tissue samples, and the exclusion of alternative causes. The process is not standardized, and clinicians must use a combination of clinical judgment and available evidence to make a diagnosis (2). Sarcoidosis generally has a favorable prognosis, with approximately 60% of cases experiencing spontaneous remission within 2 to 5 years and a 5-year survival rate of 95.4%. However, 10% of patients may develop chronic disease or serious complications. The overall mortality rate is about 5%, varying by region (3). Extrapulmonary disorders of sarcoidosis have been described with central nervous system (CNS) involvement occurring in 3 to 10% of all sarcoidosis patients, in which case the diagnosis of neurosarcoidosis (NS) is made (4).

Psychiatric symptoms and syndromes (PSS) such as depression, anxiety and fatigue, are common in sarcoidosis and other symptoms like psychosis have been described as presenting features on rare occasions (4). Fatigue and neurocognitive symptoms are overarching symptoms in sarcoidosis that – together with many other comorbidities—significantly lower the quality of life (QoL). This necessitates a multidisciplinary approach that inherently includes a psychiatric perspective on fatigue, neurocognitive symptoms and QoL (5).

In NS, PSS might occur more frequently due to the neurocognitive disruption inherent to the disease process, although it has been hypothesized that PSS in sarcoidosis may arise independently of specific neurological lesions and that other psychopathological mechanisms may be involved (5).

To the best of our knowledge, a comprehensive and systematic review on PSS in sarcoidosis is lacking. We performed a systematic review of studies reporting on PSS in patients with sarcoidosis discussing diagnostics, prevalence, pathophysiology and treatment options. A subsequent meta-analysis was performed on studies including an association analysis between sarcoidosis and PSS.

## 2 Methods

### 2.1 Search strategy and selection criteria

A systematic search was conducted across PubMed using the following search string: (neurosarcoidosis OR sarcoidosis) AND (psychiatr* OR psychosis OR psychotic OR cataton* OR cognitive OR delir* OR depress* OR manic OR mania OR anxi* OR obsessive OR compulsive). The search included studies published from January 1950 until December 2024 and was restricted to English, French and Dutch-language articles, human studies and peer-reviewed publications. The reference lists of included articles were screened for additional studies. Efforts were made to include all available studies by contacting the corresponding authors for a full-text copy of their study, if necessary.

The study was conducted in accordance with the 2020 PRISMA guidelines (6). The protocol was submitted to the Department of Medicine of the University of Antwerp prior to execution. The review was not registered.

### 2.2 Inclusion criteria and exclusion criteria

Inclusion criteria were studies and case-reports: (a) focusing on individuals with a primary diagnosis of sarcoidosis or NS; (b) reporting PSS (e.g., depressed mood, hallucinations, etc…) or syndromes (e.g., mood disorders, psychotic episodes, etc…) in patients with sarcoidosis; (c) using original data. Studies and case-reports were excluded if they: (a) Focused exclusively on organic neurological symptoms without any PSS; (b) Were duplicate publications or secondary analyses; (c) No full text was available; (d) Were written in another language than English, French or Dutch.

### 2.3 Study selection and data extraction

Two independent reviewers performed the screening. In case of disagreement, papers were retained for full-text evaluation. Where needed, a third reviewer was available in case of disagreements or uncertainties concerning eligibility. All titles and abstracts were screened to select potentially relevant case reports and studies. It was decided whether, based on the full texts of all individual studies, they fulfilled the eligibility criteria. Reports of case-series were treated as multiple individual case-reports and evaluated accordingly.

One author extracted the following data from the studies: Author, year of publication, in- and exclusion criteria, study design, study focus, study outcome, psychiatric assessment tools used, data on the interactions of PSS, treatment interventions, sample sizes of sarcoidosis patients and healthy controls, mean scores of the psychiatric assessment tools and standard deviation (SD) or interquartile range (IQR) of the psychiatric assessment tools. For the subsequent meta-analysis, the outcome measure was the OR of psychiatric symptom in patients diagnosed with sarcoidosis. In case a treatment effect was measured, only the pre-treatment data was included.

One author extracted the following data from case reports: author, year of publication, gender, age, psychiatric symptom(s) described and information concerning treatment.

The methodological quality of the included studies was assessed using the Newcastle-Ottawa Quality Assessment Scale, designed for cohort studies (7). The certainty of the evidence was appraised using the Grading of Recommendations, Assessment, Development, and Evaluation approach (8).

### 2.4 Data synthesis and analysis

All prevalence data on PSS found in the systematic review was synthesized in range, average, mean and weighted averages for each scale and psychiatric symptom if there were 2 or more datapoints.

Data in the meta-analysis were synthesized using a random-effects model to account for heterogeneity across studies. OR, and 95% confidence intervals (CI) were calculated for all included studies and for the studies in categories that allowed further analysis. We recalculated the SD from the IQR when the SD was not reported, using the formula: SD=IQR/1.35, based on the assumption of a normal distribution. Heterogeneity was assessed using the I^2^ statistic. Statistical analyses for the meta-analysis were conducted using Comprehensive Meta-Analysis version 4.0.000 (2022, Biostat, Inc.). Funnel plots and Egger’s test were used to assess publication bias. The primary meta-analysis was performed on the prevalence of all PSS in patients with sarcoidosis. Secondary analysis was performed for each psychiatric symptom if at least two studies were available.

## 3 Results

### 3.1 Systematic review

#### 3.1.1 Literature search and study selection

**Figure 1:**
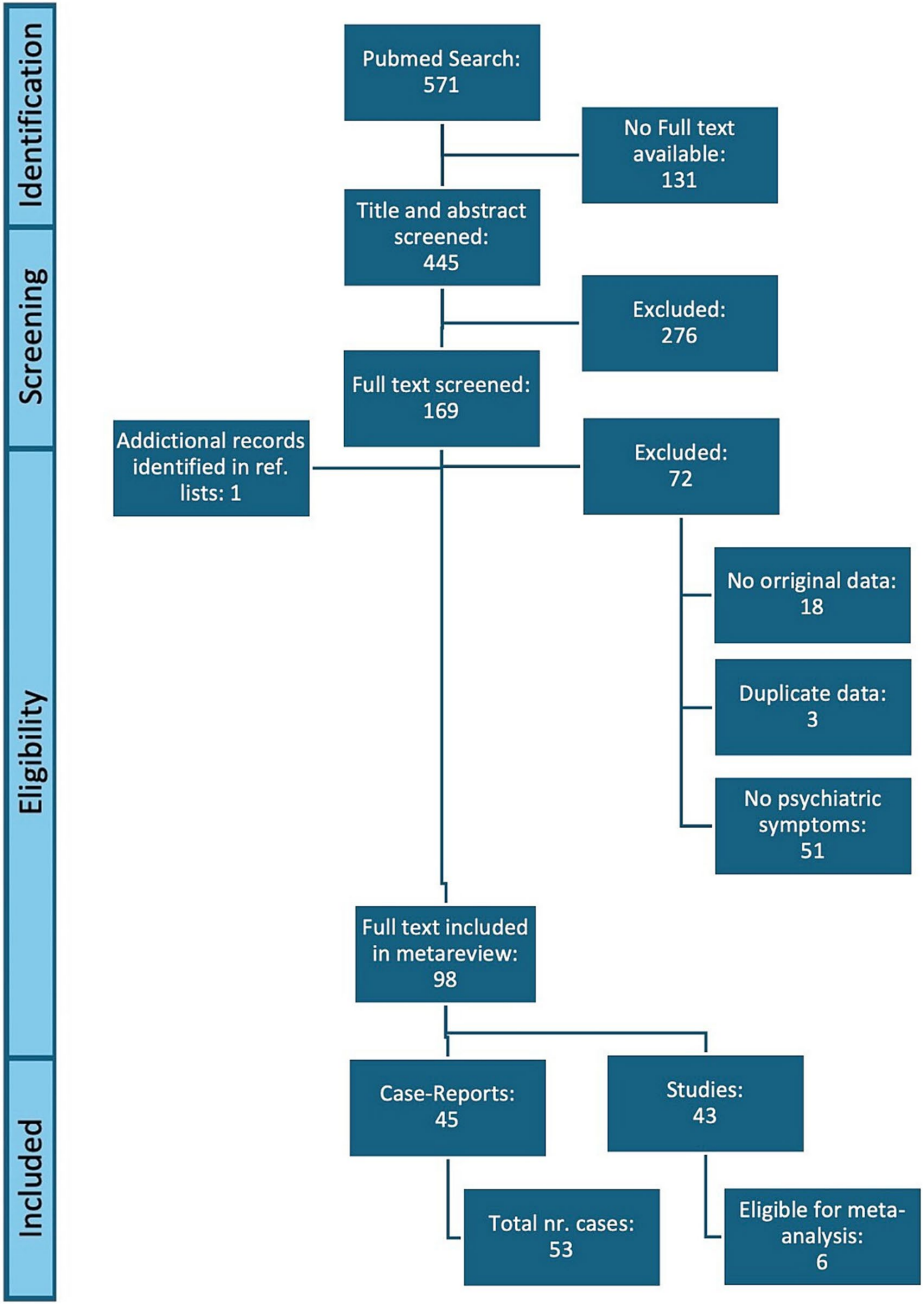
PRISMA flow diagram of literature search and study selection.

The initial search of the PubMed database yielded 571 records. The screening and exclusion process ultimately resulted in the inclusion of 43 studies reporting PSS in individuals with sarcoidosis (9–51) (Figure 1): 39 studies included patients with sarcoidosis without reporting on the possible differentiation with NS, 2 studies only included patients with NS (16, 17), 2 studies actively excluded NS (42, 46). The 45 included case-reports and case-series revealed 53 relevant cases (52–96).

Eventually, 6 studies were found eligible for subsequent meta-analysis (Table 1) (10, 23, 41, 42, 45, 48).

**Table 1:** Studies included in the meta-analysis.

Author	Year	Sample size	Controls	NOS	Grade	Tools used	Study design	Population of sarcoidosis patients
Voortman et al. (48)	2019	443	62	7	3	CESD, CFQ, FAS, STAI, PSSS, WHOQOL Bref	Cross-sectional	Patients diagnosed per WASOG guidelines
Saligan et al. (42)	2014	14	13	6	1	HAMD, FAS	Prospective cohort	Biopsy-confirmed without cardiac or neurosarcoidosis
Patterson et al. (41)	2013	62	1,005	7	3	CESD, ESS	Retrospective cohort	Patients undergoing polysomnography for suspected OSA
Elfferich et al. (23)	2010	343	343	8	2	CFQ, CESD, FAS	Cross-sectional	Diagnosis based on clinical, radiologic, and histological criteria
Antoniou et al. (10)	2006	75	30	5	2	HADS	Cross-sectional	Histologically confirmed with active disease
Spruit et al. (45)	2005	25	21	7	3	HADS, SF36	Cross-sectional	Patients with fatigue diagnosed per ATS/ERS/WASOG criteria

ATS, American Thoracic Society; CESD, Center for Epidemiologic Studies Depression Scale; CFQ, Cognitive Failure Questionnaire; ERS, European Respiratory Society; ESS, Epworth Sleepiness Scale; FAS, Fatigue Assessment Scale; HADS, Hospital Anxiety and Depression Scale; HAMD, Hamilton Depression Rating Scale; PSSS, Perceived Social Support Scale; SF36, Short Form 36 Health Survey; STAI, State–Trait Anxiety Inventory; WASOG, World Association of Sarcoidosis and Other Granulomatous Disorders; WHOQOLBref, World Health Organization Quality of Life – BREF.

#### 3.1.2 Study population, design and methodology

Study populations varied from the two biggest cohorts presented by Sikjaer et al. (*n* = 7,302) (44) and the German Sarcoidosis Society (*n* = 1,197) (14, 15, 28–30), to 17 large samples (*n* = 100–1000) and 20 smaller samples (*n* < 100). All studies either recruited from specialized centers for the treatment of sarcoidosis or gathered patient data from large epidemiological databases. Of the 43 studies 4 were interventional studies: 3 randomized controlled trials (RCT) (35, 40, 49) and 1 cohort study (38). The other 39 observational studies comprised of 30 cross-sectional studies, 7 cohort studies and 2 case–control studies. A wide variety of assessment tools was used for screening PSS. General screening for PSS with the “International Classification of Diseases, Ninth or Tenth Revision” (ICD-9/10), the “Mini International Neuropsychiatric Interview Plus” (MINI-Plus), “Symptom Checklist-90” (SCL-90) or clinical assessment was used in only 6 (14.0%) of the studies. Standardizing general screening for PSS was suggested in 10 (23.3%) studies (11, 15, 18, 24, 26, 27, 29, 33, 37, 47). PSS were the main study focus in 21 (48,8%) studies (11, 12, 14, 18, 20, 21, 24, 26, 27, 29, 31, 32, 34, 35, 38, 40, 43, 47, 48, 50, 51), of which 9 (20.9%) and 6 (14.0%) specifically investigated depression and anxiety, respectively. Fatigue and sleep-related disturbances were the main study focus in 17 (39.5%) studies (12–15, 21, 22, 24, 27, 28, 30, 35–37, 40–42, 51). Neurocognitive symptoms were a main focus in 7 (16.3%) studies (12, 16, 21, 27, 33, 46). QoL was investigated in 14 (32.6%) studies (9, 10, 13, 19, 20, 24, 26, 33, 35, 38, 40, 45, 48, 51).

#### 3.1.3 Prevalence of PSS

We found 29 (67.4%) studies mentioning prevalence in of PSS in patients with sarcoidosis (11–15, 17–20, 23–26, 28–30, 33, 34, 37–39, 41, 43–45, 47, 50, 51) (Table 2). Sample sizes varied (Range = 20–7301 avg. = 580.4 median = 148). All but 4 used self-reporting scales (26, 44, 47, 50).

**Table 2:** Studies reporting prevalence of PSS in patients with sarcoidosis.

Author	Year	Sample	Study type	Prevalence per assessment tool
Sikjaer et al. (44)	2024	7,301	Retrospective cohort	ICD10 anxiety and depression: 6.9% in sarcoidosis
Kahlmann et al. (35)	2023	99	RCT	HADS: Anxiety: 52%, Depression: 51%
Byg et al. (17)	2022	20	Prospective cohort	FAS: Fatigue: 80%, BDI: Depression: 35%
Hoth et al. (33)	2022	315	Cross-sectional	CFQ: Cognitive Difficulties: 25%
Bloem et al. (13)	2021	60	Cross-sectional	HADS: Anxiety: 12%, Depression: 8%
Froidure et al. (25)	2019	53	Prospective cohort	HADS: Anxiety: 26%, Depression: 20%; FAS: Fatigue: 41%
Sharp et al. (43)	2019	112	Cross-sectional	PHQ8: Depression: 54% (20% moderate–severe); GAD7: Anxiety: 37% (13% moderate–severe)
Lingner et al. (38)	2018	296	Prospective cohort	FAS: Fatigue: 66%; HADS: Anxiety: 30.4%, Depression: 28.4%
Moor et al. (39)	2018	210	Cross-sectional	GADSI: Anxiety 48%
Bosse-Henck et al. (15)	2017	1,197	Cross-sectional	HADS: Anxiety: 27%, Depression: 19.7%; ESS: Excessive Daytime Sleepiness: 16.5%, FAS: Fatigue: 16.4%
Ungprasert et al. (47)	2017	345	Retrospective cohort	Clinical diagnosis: Depression: 13.8%
de Boer et al. (20)	2014	56	Cross-sectional	HADS: Depression: 10.9%, Anxiety: 25.4%,
Balaji et al. (11)	2012	148	Cross-sectional	HADS: Depression: 25.7%
Hinz et al. (29)	2012	1,197	Cross-sectional	HADS: Anxiety: 26%, Depression: 18%
Elfferich et al. (24)	2011	588	Cross-sectional	FAS: Fatigue: 79.4%; CESD: Depression: 32.7%
Hinz et al. (30)	2011	1,197	Cross-sectional	FAS: Fatigue: 70% MFI: Fatigue: 68%
Korenromp et al. (37)	2011	75	Cross-sectional	CDC-criteria: Chronic Fatigue: 47%
Elfferich et al. (23)	2010	343	Cross-sectional	CFQ: Cognitive failure: 33%, FAS: Fatigue: 80%, CESD: Depression: 27%
Ireland & Wilsher (34)	2010	81	Cross-sectional	HADS: Anxiety: 32%, Depression: 23%
Goracci et al. (26)	2008	80	Cross-sectional	MINI-Plus: Major Depressive Disorder: 25%, Panic Disorder: 6.3%, Bipolar Disorder: 6.3%, Generalized Anxiety Disorder: 5%, Obsessive Compulsive Disorder: 1.3%
Westney et al. (50)	2007	165	Cross-sectional	ICD9: Depression: 13%
Spruit et al. (45)	2005	25	Cross-sectional	HADS: Depression: 38%, Anxiety: 24%
Cox et al. (19)	2004	111	Cross-sectional	CESD: Depression: 66%
Chang et al. (18)	2001	154	Cross-sectional	CESD: Depression: 60%
Wirnsberger et al. (51)	1998	64	Cross-sectional	BDI: Depression: 18%

BDI, Beck Depression Inventory; CDC-criteria, Centers for Disease Control and Prevention criteria; CESD, Center for Epidemiologic Studies Depression Scale; CFQ, Cognitive Failure Questionnaire; ESS, Epworth Sleepiness Scale; FAS, Fatigue, assessment Scale; GAD7, Generalized Anxiety Disorder 7-item scale; GADSI, Generalized Anxiety Disorder Severity Index; HADS, Hospital Anxiety and Depression Scale; ICD9/10, International Classification of Diseases 9th/10th Revision; MINI-Plus, Mini International Neuropsychiatric Interview Plus version; PHQ8, Patient Health Questionnaire-8; PSS, Perceived Stress Scale; QoL: Quality of Life.

Prevalence of depression in total was measured in 20 studies (*n* = 5,194, range = 8.0–66.0%, avg. = 29.4%, mean = 25.4%, weighted avg. = 24.9%). Results per used method differed: HADS-depression (*n* = 3,212, range = 8.0–51.0%, avg. = 24.3.%, mean = 21.5%, weighted avg. = 21.0%), CESD (*n* = 1,196, range = 27.0–66.0%, avg. = 46.4%, mean = 46.4%, weighted avg. = 37.7%), BDI (*n* = 84, range = 18.0–35.0%, avg. = 26.5%, mean = 26.5%, weighted avg. = 22.0%) and non-self-reporting tools: MINI-Plus, ICD9 and clinical diagnosis (*n* = 590, range = 13.0–25.0%, avg. = 17.3%, mean = 13.8%, weighted avg. = 15.1%).

Prevalence of anxiety was assessed in 12 studies (*n* = 3,466, range = 12.0–52.0%, avg. = 29.4%, mean = 26.5%, weighted avg. = 28.7%), primarily using HADS-anxiety (n = 3,064, range = 12.0–52.0%, avg. = 28.3%, mean = 26.0%, weighted avg. = 27.5%); exceptions included three studies using Generalized Anxiety Disorder 7-item Scale (GAD-7) (37.0% *n* = 112), Generalized Anxiety Disorder Severity Index (GADSI) (48.0% *n* = 210) and MINI-Plus (12.6% *n* = 80) respectively (*n* = 402, range = 12.6–48.0%, avg. = 32.5%, mean = 37.0%, weighted avg. = 37.9%). The different MINI-Plus-diagnoses for anxiety (generalized anxiety disorder, obsessive compulsive disorder and panic disorder) were combined into a single percentage of 12.6%. Sikjaer et al. (44) reported a hazard ratio of anxiety and depression over a period of 15 years of 6.9% in 3701 patients from the Danish National Patient Register with newly diagnoses sarcoidosis versus 4.9% in 26,145 matched controls, mainly identifying patients on the basis of prescription-data; see also: limitations.

Neurocognitive symptoms occurrence was measured in only 2 studies, using the Cognitive Failures Questionnaire (CFQ) (*n* = 658, range = 25.0–33.0%, avg. = 29.0%, mean = 29.0%, weighted avg. = 29.2%).

The prevalence of fatigue was measured in 7 studies using the Fatigue Assessment Scale (FAS) (n = 3,694, range = 16.4–80.0%, avg. = 61.8%, mean = 70.0%, weighted avg. = 54.4%).

Excessive daytime sleepiness was reported on in 3 studies using the Epworth Sleepiness Scale (ESS): 16.5% (*n* = 1,197, ESS > 16) (15), 50.0% (*n* = 1,197, ESS > 10) (28), 60.0% (*n* = 62, ESS > 10). Since a cutoff of ESS > 10 is considered the standard threshold for excessive daytime sleepiness, the following statistics were calculated using only the latter two studies (*n* = 1,259, range = 50.0–60.0%, avg. = 55.0%, mean = 55.0%, weighted avg. = 50.5%).

As to sleep disturbances (*n* = 1,281 range = 25.0–54.0% avg. = 39.5%, mean = 39.5%, weighted avg. = 26.9%), 54% of sarcoidosis patients reported frequent and occasional sleep disturbance compared to only 17% of healthy controls (*p* < 0.0001) in the study by Benn et al. (12). Insomnia defined as a Pittsburgh Sleep Quality Index of more than 10 was reported in 25% (14).

One study reported on the relative risk of developing schizophrenia (n = 345, HR = 2.15, CI = 0.39–11.76) and substance abuse disorder (*n* = 345, HR = 1.55 CI = 0.44–5.49) over 5 years in sarcoidosis (47). However, neither association reached statistical significance.

We found no comprehensive reporting of other PSS.

#### 3.1.4 Interactions among PSS in sarcoidosis

PSS in sarcoidosis have an assumed multifactorial etiology. In our systematic review we could only find anecdotal evidence to support this in 8 (18.6%) studies.

One study mentioned an higher relapse rate of neurocognitive symptoms in patients with parenchymal lesions (*n* = 9) (16). Another shows a difference with fMRI in brain activity in the absence of apparent neurological lesions when comparing patients with sarcoidosis with and without chronic fatigue (*n* = 30) (36).

Overall fatigue was a key symptom in 5 (11.9%) of the studies and has been linked to neurocognitive symptoms and depressive symptoms (*n* = 292) (27). When compared to controls, individuals with sarcoidosis consistently exhibit higher levels of fatigue and depression (*n* = 27) (42). Notably, fatigue often persists even in the context of clinical remission and is associated with psychological distress (n = 75) (37).

Obstructive sleep apnea (OSA) is very prevalent in sarcoidosis patients and several clinical manifestations like PSS are common to both OSA and sarcoidosis (97). Although abnormal lung function in sarcoidosis may contribute to OSA, sarcoidosis remains independently associated with excessive daytime sleepiness (n = 62) (41).

Physical fitness and particularly muscle strength showed a similar three-way association with either sarcoidosis and PSS in small cohorts (*n* < 112) (37, 42, 43, 45).

#### 3.1.5 Treatment interventions

Research specifically addressing treatment interventions for PSS in patients with sarcoidosis was found to be scarce, with only 8 studies (18.6%) reporting on this topic. A RCT (*n* = 99) showed online mindfulness-based cognitive therapy significantly improved fatigue, anxiety and depression in sarcoidosis patients (35). A RCT (*n* = 18) showed 12 week exercise training significantly improved QoL, anxiety and fatigue (40). One study (*n* = 296) reported that fatigue, anxiety, depression, and QoL was significantly improved in sarcoidosis patients after a 3 week pulmonary rehabilitation program (38). Another study, however, failed to observe a significantly increase in daily life physical activity in patients with chronic sarcoidosis 10 months after a 2 months pulmonary rehabilitation suggesting the need for long-term behavioral interventions (49). A retrospective cohort study (*n* = 9) compared different treatment strategies of NS and among others discussed their effect on cognition with mixed results (16). A prospective cohort study (*n* = 20) showed that after one-year immunosuppression 75% of patients experienced important clinical improvement but no difference in fatigue and depression (17). A cross-sectional study (*n* = 343) showed anti-TNF-alpha treatment improved neurocognitive function and reduced fatigue in some patients, but effects varied (23). The role of physical rehabilitation has been suggested as a way to reduce PSS (42, 45). Sikjaer et al. (44) reported on 80.5% of patients with sarcoidosis taking antidepressants without having a diagnosis of anxiety or depression.

#### 3.1.6 Characteristics of the included case reports

The 53 included cases revealed a gender distribution of 56.6% female cases and large variation of age (range = 16–79 years, avg. = 44.6 years mean = 41 years). The following PSS were reported: Neurocognitive symptoms (54.7%), psychosis (39.6%), mood disturbances (24.5%), delirium (17.0%), behavioral changes (15.1%), bipolar symptoms (11.3%), anxiety (9.4%), and catatonia (5.7%). We found no cases reports on addiction or eating disorders in patients with sarcoidosis. PSS were the presenting feature of sarcoidosis in 15.1% of all case reports.

Case reports detailing treatment strategies predominantly emphasized supportive therapies for sarcoidosis, supplemented by symptomatic management of comorbid PSS. Treatment for PSS was mentioned in 28 cases among which: antipsychotics (71.4%), antidepressants (32.1%), anti-epileptics (28.6%), benzodiazepines (25.0%), lithium (7.1%), electroconvulsive therapy (7.1%) and psychotherapy (7.1%).

In 80% of these cases, psychiatric and physical symptoms of sarcoidosis followed a synchronous course, with both improving or worsening in parallel according to the response to the primary treatment of sarcoidosis.

### 3.2 Meta-analysis

#### 3.2.1 Study characteristics

The meta-analysis incorporated 6 studies from the systematic review investigating the association between sarcoidosis and PSS (10, 23, 41, 42, 45, 48). These studies collectively assessed 1,119 patients diagnosed with sarcoidosis and 2,605 healthy controls. The studies employed 11 different psychiatric assessment tools to evaluate depressive symptoms, anxiety, neurocognitive symptoms, and fatigue (Table 1). While one study explicitly excluded individuals with NS from its analysis (42), the remaining five studies did not specify differentiation between sarcoidosis and NS. Sample sizes varied across studies, ranging from small-scale cohorts to larger population-based analyses. The diagnostic methodologies and psychiatric screening tools differed significantly. The methodological quality of the included studies, as evaluated by the Newcastle-Ottawa Scale, was deemed acceptable (Table 1).

#### 3.2.2 Association of PSS and sarcoidosis

**Figure 2:**
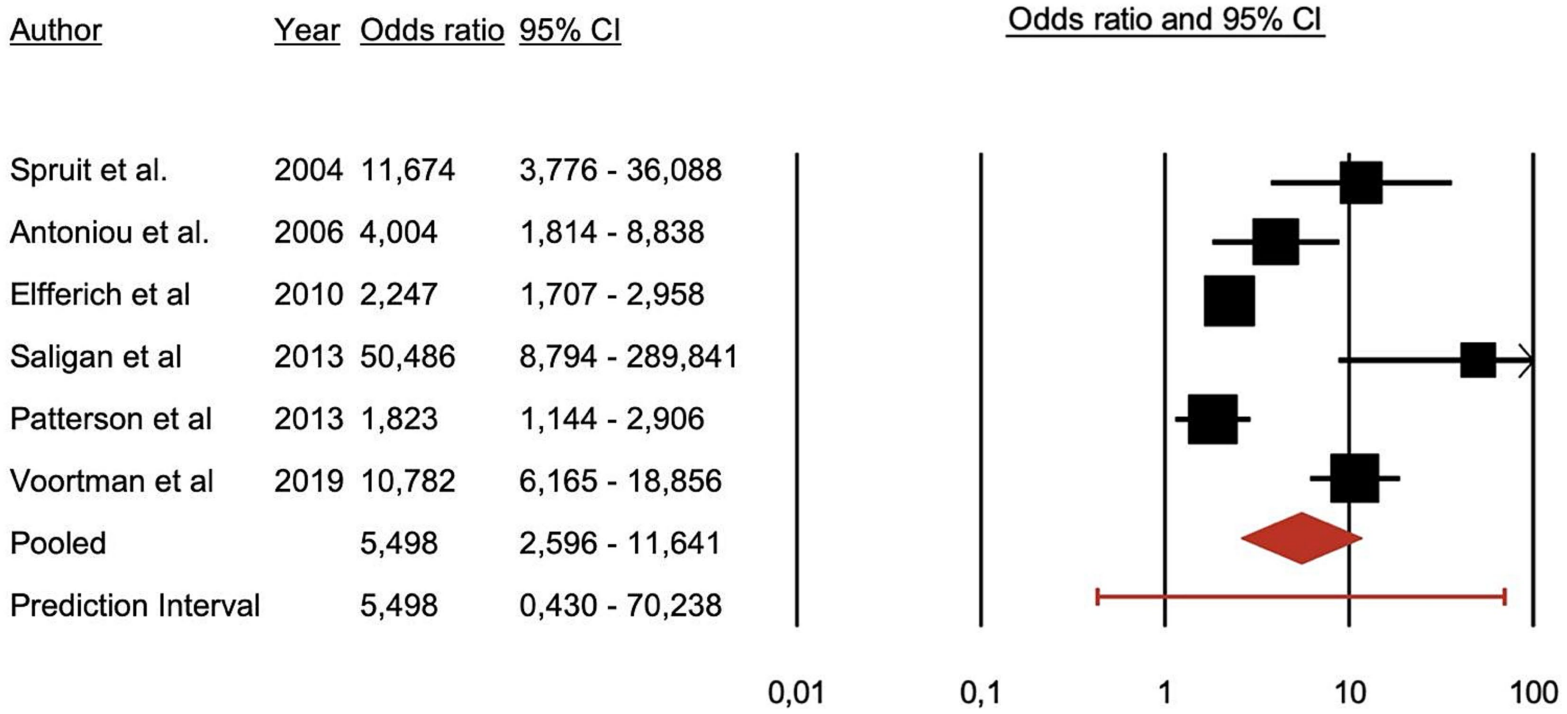
Forest plot illustrating the association between psychiatric symptoms and syndromes and sarcoidosis.

All included studies reported a higher prevalence of PSS among sarcoidosis patients compared to control groups. The primary overarching analysis of the meta-analysis demonstrated that patients with sarcoidosis exhibit significantly more PSS compared to healthy controls (OR = 5.498, 95% CI = 0.430–70.238; *p* < 0.001) (Figure 2).

Secondary analyses by symptom category demonstrate a significant association of sarcoidosis with depressive symptoms and fatigue. Anxiety, and neurocognitive symptoms were also associated with sarcoidosis although with less available evidence as these two categories had only two studies each in their analysis. Depressive symptoms: patients = 180, controls = 1,069, OR = 4.855, z = 2.401, *p* = 0.016. Anxiety disorders: patients n = 104, controls n = 51, OR = 6.372, z = 5.600, *p* < 0.001. Neurocognitive symptoms: patients = 551, controls = 405, OR = 2.796, z = 3.911, p < 0.001. Fatigue: patients = 284, controls = 1,080, OR = 20.231, z = 2.868, *p* = 0.004.

#### 3.2.3 Sensitivity analyses and certainty of evidence

**Figure 3:**
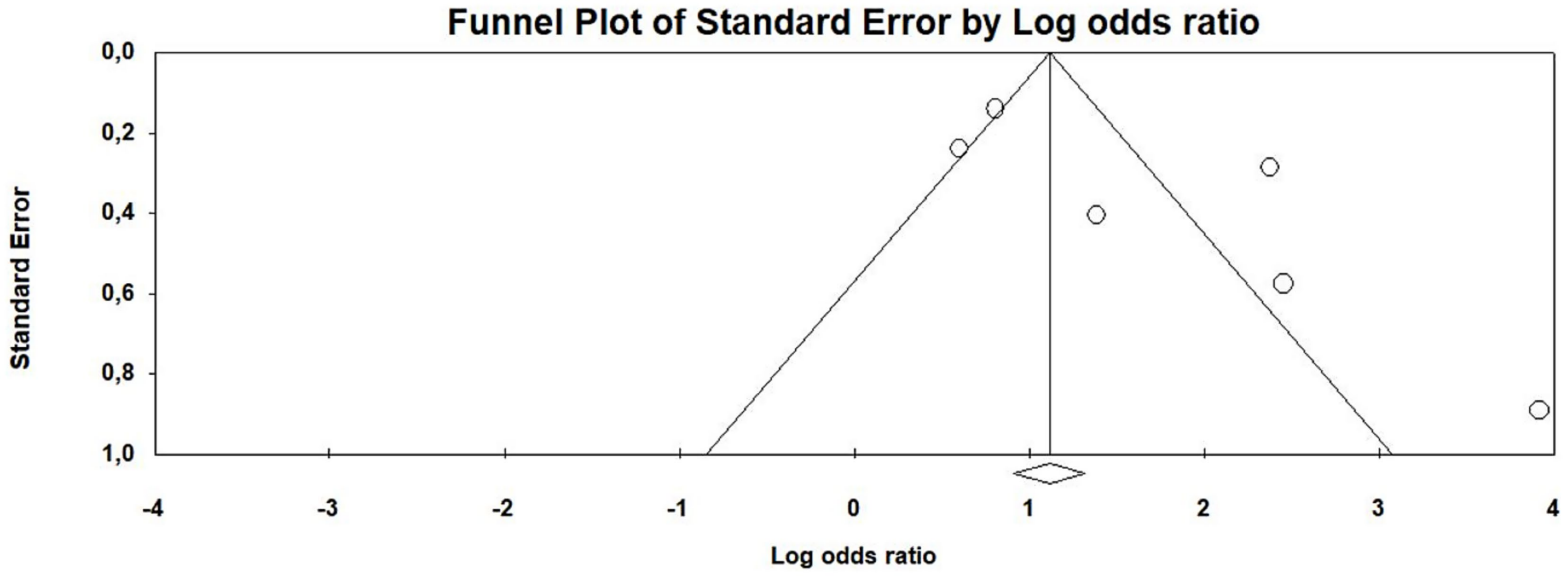
Funnel plot of publication bias of the meta-analysis.

Funnel plots were examined to assess potential publication bias in the meta-analysis. The plots indicate evidence of publication bias, as studies with positive and significant results appear to be overrepresented (Figure 3). This observation is supported by Egger’s test, which yielded a statistically significant result (t = 2.44, *p* = 0.0371), further suggesting the presence of publication bias.

The certainty of evidence of our meta-analysis was determined as low to moderate with a weighted average GRADE score of 2.54 on a scale of 1 to 4. This is mainly due to the observational study design and the publication bias detected.

## 4 Discussion

### 4.1 Main findings

In this systematic review on PSS in patients with sarcoidosis, prevalence of PSS varied widely depending on the study design with a weighted average of 24.9% for depression, 28.7% for anxiety, 29.2% for neurocognitive symptoms, 54.4% for fatigue, 50.5% for excessive daytime sleepiness (ESS > 10) and 26.9% for sleep disturbances and insomnia.

The subsequent meta-analysis of 6 eligible studies confirms that patients with sarcoidosis have an increased risk of developing PSS when compared with healthy controls (OR = 5.498, CI = 0.430–70.238, *p* < 0.001). Depressive symptoms and fatigue were most reported and demonstrated the strongest associations (resp. OR = 4.855, z = 2.401, *p* = 0.016 and OR = 20.231, z = 2.868, *p* = 0.004). Associations with anxiety and neurocognitive symptoms were also observed.

The case reports revealed a diagnostic diversity not reflected in study populations, pointing to heterogeneity and the need for a more systematic use of comprehensive psychiatric assessment tools.

### 4.2 Etiopathogenesis

Multiple principal yet interplaying mechanisms have been proposed to underlie sarcoidosis-related PSS including neuroinflammatory processes, immune-mediated pathways, disturbances in calcium-metabolism, disruption of neuronal functioning and psychosocial factors (88). However, as mentioned in the results, the evidence on the etiopathological factors is still scarce and mainly consists of data on interacting and mediating factors.

Direct inflammatory involvement of the CNS may lead to the formation of non-caseating granulomas in the parenchyma or meninges, triggering the release of pro-inflammatory cytokines such as interleukin-6 (IL-6), tumor necrosis factor alpha (TNF-*α*), and interferon-gamma (IFN-*γ*), which disrupt neurotransmitter systems and impair limbic and frontal lobe structures (88). Interestingly, increases in pro-inflammatory markers such as IL-6, TNF-a and IFN-g have been associated to mood and psychotic symptoms (98).

In addition, blood–brain barrier disruption may facilitate immune cell infiltration into the brain. Inflammatory processes and an altered immune response have been implicated in the pathogenesis in sarcoidosis, other CNS inflammatory and auto-immune disorders, as well as psychosis, delirium, and depression (99, 100). A shared feature across sarcoidosis and psychosis, particularly in later stages, appears to be a blunted cellular immune response combined with an over-activated type-2 immune response (88). IL-6, IL-8, and TNF-*α* concentrations have shown to be elevated with the kynurenine pathway in depression and schizophrenia (101). Pro-inflammatory cytokines can stimulate the activity of indoleamine 2,3-dioxygenase and lead to tryptophan deficiency and thus a reduction in the production of 5HT and melatonin, with a shift to the production of kynurenine and other neurotoxic tryptophan-derived metabolites (101). These higher values could then explain the white and grey matter degeneration in schizophrenia and in sarcoidosis patients in granuloma formation, with the possible development of fibrosis in the CNS This supports the hypothesis of a shared etiopathogenic link between sarcoidosis and psychosis (88).

Hypercalcemia is a recognized complication in sarcoidosis, occurring in about 6–18% of cases. This condition is often due to increased production of 1,25-dihydroxyvitamin D by activated macrophages within granulomas, leading to enhanced calcium absorption. Hypercalcemia can contribute to neurological symptoms, including encephalopathy, which may present with neurocognitive symptoms and altered mental status (102, 103).

As previously noted, up to 10% of patients with sarcoidosis develop some form of NS with neurological lesions present in half of these cases, potentially disturbing neurological functioning on a systemic level (4). The implied multilevel etiology suggests that PSS may occur in sarcoidosis both with and without overt neurocognitive disruption caused by disease-related lesions. According to Focke et al. (104), patients with cerebral vasculitis related to NS are significantly more likely to present with neurological symptoms, including cognitive and/or behavioral changes. Notably, the CNS involvement may be asymptomatic and the diagnosis of NS is often complicated by the heterogeneous disease presentation and the absence of specific diagnostic tests (5).

### 4.3 Psychosocial suffering and fatigue

Psychosocial suffering and fatigue is considered a possible cause and consequence of PSS in sarcoidosis. The chronic burden of sarcoidosis, accompanied by persistent fatigue and pain, may cause chronic somatic and psychosocial stress and reactive depression and anxiety, with a severe impairment and impact on social and occupational functioning may further enhance these complaints (105). In the study by Hinz et al. (29), the number of affected organs, the number of concomitant diseases and the degree of dyspnea significantly predicted anxiety and depression scores. Furthermore, sleep apnea, restless legs syndrome, and arterial hypertension were associated with anxiety as well as depression (29). Moreover, additional predictors for depressive symptoms were female sex, decreased access to medical care, and increased dyspnea (18). Furthermore, sarcoidosis patients with PSS showed a higher odds for emergency department visit in the previous 6 months and a worse health-related quality of life, compared with patients without PSS (43).

Fatigue in sarcoidosis is a multifactorial symptom influenced by a complex interplay of physiological and psychological factors and seems, in part, intrinsic to the disease. This impairs daily coping mechanisms and disrupts social interactions in patients that are often of a younger age, making them less resilient to this often chronic and burdensome disease (10). Fatigue has been linked to neurocognitive and depressive symptoms, small fiber neuropathy, and dyspnea (27, 41, 42), as well as sleep disturbances (12). Fatigue often persists even in the context of clinical remission and is associated with psychological distress, diminished health status, decreased physical activity and muscle weakness (37, 42, 43, 45). Which are all features also associated with chronic fatigue syndrome (106). Muscle involvement in sarcoidosis is common and physical fitness has been shown to be associated to PSS and fatigue (37, 42, 43, 45).

Sleep disturbance significantly correlated with fatigue, depression, cognitive dysfunction, and poor quality of life, independent of disease severity (12). Poor sleep quality is highly prevalent in patients with sarcoidosis and strongly associated with fatigue, anxiety, and depression (14). OSA is very prevalent in sarcoidosis patients and several clinical manifestations like PSS are common to both OSA and sarcoidosis (97). Although abnormal lung function in sarcoidosis may contribute to OSA, sarcoidosis remains independently associated with excessive daytime sleepiness, again emphasizing a multifactorial etiology (*n* = 62) (41).

All the above supports other research indicating that psychosocial suffering and fatigue – like PSS – in sarcoidosis requires multimodal approach and individual care strategies (107).

### 4.4 PSS as an effect of sarcoidosis treatments

Commonly used treatments for sarcoidosis have been associated with PSS. Corticosteroids, commonly have been associated with psychiatric disturbances such as depression, mania, and psychosis. The overall incidence of psychiatric symptoms from corticosteroids occurs in approximately 25% of patients (108). As it can even increase suicidal behavior patient education is warranted (109). Corticosteroids are also known to induce CYP3A4 activity although with extensive intersubject variability, lowering plasma concentrations of many antidepressants and antipsychotics (110). Anti-TNF-alpha therapy can, in rare cases, induce psychiatric side effects including mania, hypomania, and psychosis, even in patients without prior psychiatric history (111). These effects are generally reversible upon discontinuation of the drug (112).

### 4.5 Psychiatric screening within a multidisciplinary framework

As discussed PSS in sarcoidosis may occur in sarcoidosis with and without overt neurocognitive disruption caused by disease-related lesions (5). Comparing diagnostic diversity between studies and case reports, we found a notable discrepancy between the high diversity of PSS that have been identified in case reports on one hand and the limited focus of diagnostic and screening tools used in studies on sarcoidosis on the other. Indeed, whereas 39.6% of the cases reported psychotic symptoms, only 5 (11.6%) of the included studies actually employed psychiatric screening capable of capturing such symptoms. A higher association was investigated by Ungprasert et al. (47) but did not reach statistical significance. Psychotic symptoms rarely become clinically apparent in the early stages of the disease, because of the rather atypical clinical presentation with mainly affective symptoms or fatigue, ubiquitous in sarcoidosis (113). Notably, studies relying on retrospective medical file analysis reported lower depression prevalence—only up to 13.8% (44, 47, 50)—highlighting the importance of active screening.

Taken together, these findings add to existing literature highlighting the need for standardized psychiatric screening in sarcoidosis research and care using dedicated assessment tools. While routine psychiatric screening may not be necessary for many sarcoidosis patients without chronic or severe disease, it should nonetheless be applied with a low threshold when somatic or psychological distress is suspected; ideally imbedded in a multidisciplinary framework, for example through a proactive, team-based consultation-liaison approach that fosters collaboration between psychiatry and other specialties (114, 115). This allows coordinating internal medicine specialists to better mediate between disciplines, and ensuring a comprehensive, patient-centered management strategy integrating the complex somatic and psychological aspects of sarcoidosis (107).

### 4.6 Treatment of PSS in sarcoidosis

Evidence based treatment strategies for PSS in sarcoidosis remain limited, with only a few studies addressing potential interventions (35, 38, 40, 49). Overall, the current practice is limited to supportive therapies for sarcoidosis, added by symptomatic management of comorbid PSS as needed. Pharmacological treatment of depression and anxiety in sarcoidosis includes SSRIs or SNRIs as first-line options, with a recommended treatment duration of at least 6–12 months to reduce relapse risk (44).

Cognitive Behavioral Therapy is the preferred psychotherapeutic approach for depression and anxiety, particularly for patients experiencing fatigue or maladaptive coping patterns. Combining pharmacotherapy with psychotherapy yields superior outcomes compared to either modality alone (43). Psychotherapeutic interventions have been shown to enhance physical rehabilitation, and vice versa in small studies, encouraging further research in multidisciplinary treatment approaches (35, 38, 40, 49). However, a recent meta-analysis pulmonary rehabilitation failed to show any added value in treating fatigue (116). The European Respiratory Society’s clinical guidelines do, however, still formulate conditional recommendations for treating fatigue in sarcoidosis—stating low quality of evidence—regarding the use of pulmonary rehabilitation program and/or inspiratory muscle strength training for 6–12 weeks, and, if deemed useful, a trial with d-methylphenidate or armodafinil (117).

No additional therapies specific to certain PSS in sarcoidosis were identified necessitating already established consultation-liaison psychiatry guidelines for the management of these presentations. Case reports seemed to show that the most promising improvements in PSS tend to follow a successful therapeutic response to sarcoidosis. It remains clear however that untreated PSS are detrimental to not only QoL but also—to an unknown extent - to clinical progression of sarcoidosis, whether those PSS are reactive or comorbid.

Additionally, patient and healthcare provider education play a crucial role in accurately assessing symptoms and their impact (19, 31, 32, 34), ensuring a more comprehensive understanding and management of PSS in sarcoidosis again highlighting the importance of an integrated treatment strategy.

### 4.7 Limitations

In general, research on sarcoidosis presents significant challenges. The disease exhibits a heterogeneous presentation with variable severity and an unpredictable course. Diagnostically, there is no golden standard, and it remains a diagnosis of exclusion with a prolonged diagnostic process and requiring histological confirmation (1).

Diagnostic variance also presents a challenge for research on PSS. For instance, in the study by Sikjur et al. (44), more than 80% of the patients with sarcoidosis suffering from anxiety and/or depression in their cohort were defined as more than one redeemed prescription of antidepressants within 1 year, instead of a clear diagnosis based on criteria (44).

Many of the studies are of cross-sectional design, so a statement about causal relationships is not possible. The majority of data are based on the subjective self-reporting questionnaires.

The primary meta-analytic outcome showed a significant association, but the wide confidence interval (OR = 5.498, 95% CI = 0.430–70.238) reflects uncertainty. This likely stems from heterogeneity and small sample sizes and underscores the need for more robust studies to confirm these findings.

Differentiating between sarcoidosis and NS is complex. Research on PSS in sarcoidosis was largely constrained to observational studies. This may contribute to the underreporting of NS; however, as previously discussed, its distinction may be questioned in the context of PSS. The design of the individual studies is highly heterogeneous, with generally a small sample size and a lack of control for important confounding factors. As a consequence, the number of studies and the sample size of the meta-analysis was relatively small. Furthermore, the available data also did not permit adjustment of the results to account for a potential subpopulation of patients with NS, which may have influenced the outcomes.

Research on PSS in sarcoidosis focusses on depression, anxiety and fatigue. For example, less research has been done on neurocognitive symptoms, sleep and psychotic symptoms. Notably, we did not assess anorexia, as this symptom it is often associated with the disease and the treatment drugs can cause gastro-intestinal side effects and reduced appetite (5).

Finally, the methodological quality of included case-reports was not evaluated using a quality assessment tool.

## 5 Conclusion

Despite methodological variations across studies we describe in our systematic review a consistent trend indicating patients with sarcoidosis exhibit a significant higher prevalence of PSS. Our meta-analysis confirmed the significance of this higher prevalence when compared to healthy controls. Secondary analyses by symptom category also showed a significant association of sarcoidosis with depressive symptoms and fatigue. Anxiety, and neurocognitive symptoms were also associated with sarcoidosis although with less available evidence. The etiopathogenesis is multifactorial and complex with among others. PSS interacting with each other and treatment options for sarcoidosis possibly causing PSS themselves.

Nonetheless, research in this area remains limited by methodological heterogeneity, small sample sizes, and a lack of interventional studies. Standardized psychiatric screening tools should be integrated into routine sarcoidosis management protocols, and future research should focus on targeted therapeutic interventions and interdisciplinary care models.

## Data Availability

The original contributions presented in the study are included in the article/supplementary material, further inquiries can be directed to the corresponding author.
